# Advanced human embryo research beyond the 14-day limit: A bioethical perspective from the Muslim world

**DOI:** 10.1016/j.stemcr.2024.06.008

**Published:** 2024-07-25

**Authors:** Mohammed Ghaly, Essam M. Abdelalim

**Affiliations:** 1 Research Center for Islamic Legislation & Ethics (CILE), College of Islamic Studies, Hamad Bin Khalifa University (HBKU), Qatar Foundation, Education City, Doha, Qatar; 2 College of Health and Life Sciences, Hamad Bin Khalifa University (HBKU), Qatar Foundation, Education City, Doha, Qatar; 3 Laboratory of Pluripotent Stem Cell Disease Modeling, Translational Medicine Division, Research Branch, Sidra Medicine, Doha P.O. Box 26999, Qatar; 4 Diabetes Research Center, Qatar Biomedical Research Institute (QBRI), Hamad Bin Khalifa University (HBKU), Qatar Foundation (QF), Doha PO Box 34110, Qatar

## Abstract

Advancements in *in vitro* human embryo research prompt a reconsideration of the 14-day rule, highlighting the integration of global religious perspectives, particularly Islam. Through analyzing classical Muslim scholars’ perspectives and modern interdisciplinary Islamic bioethical discussions, we advocate extending the 14-day limit to at least 40 days, with specified conditions.

## Introduction

This article highlights the overlooked Islamic perspectives on the 14-day rule in international guidelines for human embryo research, predominantly shaped by Western, secular traditions. Despite their global influence, these guidelines constrain Muslim-majority countries, where Islamic perspectives hold more permissive views. Recent developments underscore the urgent need for a more inclusive discourse, especially as Muslim countries increasingly invest in research institutions and collaborate with their Western counterparts. Religiously rooted bioethical perspectives are also gaining prominence, exemplified by the 17th World Congress of Bioethics in Doha, Qatar, which took place on 3–6 June 2024. This event marks the first edition hosted in the Arab world and the entire Middle East, centered on the theme “Religion, Culture, and Global Bioethics” (https://wcb.cilecenter.org/wcb#/?lang=en).

Significant strides in human embryo research and discussions reflect centuries-old interactions between science and ethics, shaping our understanding of the human body and the broader universe. Figures like Aristotle (d. 322 BCE), Avicenna (1037), Maimonides (1204), and Aquinas (d. 1274) have profoundly shaped both developmental biology and ethical considerations. Muslim scholars have similarly engaged critically with these interdisciplinary sources, verifying religio-ethical positions on human embryos (Ghaly, 2014). Amid shifting paradigms in modern biomedical advancements, particularly embryo research, the Muslim world’s discussions have consistently integrated Islamic values, advocating for nuanced approaches rooted in pre-modern Islamic scholarship.

## Human embryo in the Islamic moral tradition

To explore contemporary Islamic perspectives on embryo research and elucidate their methodological foundations and modes of reasoning, this article begins with an overview of pertinent pre-modern discussions, followed by an examination of contemporary perspectives. Drawing from dominant positions in classical and modern discourse, we then propose an engaging perspective that integrates these historical and current viewpoints.

### Pre-modern discourse

Analyses from early Muslim scholars indicate that an *in vivo* embryo was typically perceived as part of the unseen world (*ghayb*), knowledge of which is exclusive to God and primarily conveyed through the Quran and Sunna. They also acknowledged empirically verified medical information as a valid source of knowledge, aiming to reconcile insights from diverse sources, grounded in the belief that God is the ultimate source of both medical knowledge and religious scripture.

The moral standing of the human embryo was not fixed but subject to change based on three key factors.

#### Place

Some canonical Prophetic traditions explicitly describe the embryo as a fusion of two fluids, literally waters (in Arabic *māʾān*), one from the male and the other from the female. Based on the medical knowledge available at the time, pre-modern Muslim scholars viewed a woman’s womb as the sole environment where this mixture of fluids (fertilized egg) could exist and develop until birth. Grounded in these scriptural references and medical understanding, Muslim scholars concurred that the initial morally significant status of a human embryo begins with its implantation in the womb. While male and female fluids could exist separately outside the womb, neither had the potential to independently progress to the stage of a viable embryo or a subsequently born child (Ghaly et al., 2020; Musallam, 1983).

#### Age

The Quran and Sunna contain numerous references reinforcing the concept of distinct developmental stages of the embryo inside the womb. The prevailing view among Muslim scholars is that these references indicate the unborn progresses through three primary phases: *nuṭfa* (sperm-drop) for 40 days, *ʿalaqa* (a clot of congealed blood) for an additional 40 days, and *muḍgha* (a lump of flesh) for the subsequent 40 days. Upon completing these stages, totaling 120 days, the human soul gets breathed into the fetus. This marks a significant moral juncture, as unanimously agreed among Muslim scholars, permitting the termination of pregnancy only if it poses a risk to the mother’s life. For pregnancies younger than 120 days, some scholars show leniency, allowing termination even without compelling reasons. Conversely, others emphasize the pre-ensoulment phase, asserting that the embryo/fetus acquires human form (*takhalluq*), which is recognizable through the development of limbs. This latter group assigns a higher moral status to the early embryo, advocating for increased protection (Ghaly, 2012; 2014).

#### Stakeholders

In determining the moral status of the unborn, Muslim scholars identified three primary stakeholders related to the embryo: God, the Creator; the mother, who provides the female fluid (egg) and the uterus; and the father, who contributes the male fluid (sperm). Regarding God, scholars agree that plans for procreation must align with His commandments regulating sexual relations, emphasizing marriage as the fundamental context for childbirth within a husband-wife relationship. Furthermore, God’s prohibition of unjustified harm to humans extends to ensouled fetuses. In considering the parents, scholars generally accord greater weight to the mother’s perspective. However, the father’s role remained significant due to the established marital relationship, which entails mutual rights and obligations, and his material contribution to the embryo’s formation through the male fluid (sperm) (Brockopp, 2003).

### Contemporary discussions

Unlike Western, predominantly secular, deliberations, the Muslim world’s discussions have consistently integrated their religious value system (Table 1). Institutionalized since the early 1980s, interdisciplinary dialogues involve numerous religious scholars and biomedical scientists. Leading institutions include the Islamic Organization for Medical Sciences in Kuwait, the International Islamic Fiqh Academy in Jeddah, and the Islamic Fiqh Council (IFC) in Mecca, Saudi Arabia. These discussions bridge pre-modern Islamic scholarship with contemporary insights, actively engaging with Western discourse (Table 1).

**Table 1:** Embryos as research subjects: Mapping ethical perspectives

Advocates	Position	Reason
(1) (West) Germany	Categorical prohibition	Historical reasons related to the Nazi violations of human dignity during World War II
(2) Catholic Church	Categorical prohibition	- Religious perception of indivisible and unranked human dignity from the moment of fertilization - Thus, *ex vivo* embryos are considered full-fledged members of the human species
(3) Islamic minority position (mainly biomedical scientists)	Categorical prohibition	- Religious reasoning supporting West Germany’s sensitivity to human dignity - Scientific rationale asserting that human dignity is linked to the moment of fertilization, coinciding with having the complete human genome
(4) Islamic minority position (mainly religious scholars)	Unconditional permissibility	Religious reasoning advocating for an indivisible human dignity tied to the metaphysical process of ensoulment, occurring within the uterus after 120 days of pregnancy
(5) Mainstream Western position (Warnock Committee and EU regulations)	Conditional permissibility	- Scientific reasoning correlating human dignity with the emergence of the primitive streak around day 14 of pregnancy. - Recognizing the public benefit arising from research on human embryos
(6) Islamic majority position (religious scholars and biomedical scientists)	Conditional permissibility	Religio-scientific reasoning advocating for a nuanced hierarchy of human dignity, contingent on multiple factors, including (a) embryo’s location, inside or outside the uterus, (b) its age, calculated from the moment of fertilization, and (c) permission of involved stakeholders.
Proposed Islamic perspective on reassessing the 14-day limit	Conditional permissibility, up to at least 40 days	Religio-scientific reasoning, as detailed in (6), under the following conditions: ^∗^ Embryos are not obtained from religiously prohibited sources, such as forbidden abortions. ^∗^ Informed consent is obtained from the parents of the embryo. ^∗^ Embryo research is justified by scientific and ethical grounds and endorsed by an ethics committee. ^∗^ Embryos are not the subject of financial contracts (no selling or buying). ^∗^ Embryo’s age is younger than 120 days, following the majority position outlined in (6), or 40 days or younger, as agreed upon in (4) and (6).

Two minority perspectives, prohibitionist and permissivist, present opposing views on the moral status of embryos (Table 1). Prohibitionists argue for granting full moral status to embryos at fertilization, opposing embryo research and advocating the natural demise for *in vitro* fertilization (IVF) surplus embryos. West Germany’s strict 1990 “Embryo Protection Act” exemplifies this stance, hailed by prohibitionists as a courageous decision reflecting principles found in Islamic and humanistic consciences. Permissivists, conversely, draw on scriptural references, positing ensoulment after 120 days as the beginning of human life and its associated dignity. This viewpoint permits a wide range of options, including research on *ex vivo* IVF surplus embryos (Ghaly, 2012).

The majority position, however, stood out for its recognition of the nuances inherent in pre-modern Islamic scholarship and awareness of the ethical implications arising from modern biomedical advancements. Criticizing the prohibitionist stance, it noted its oversight of these nuances and its alignment with the Catholic position that was perceived as excessively stringent (*mutashaddid jiddan*) (Table 1).

### Toward a systematic and principle-based perspective

**Figure 1:**
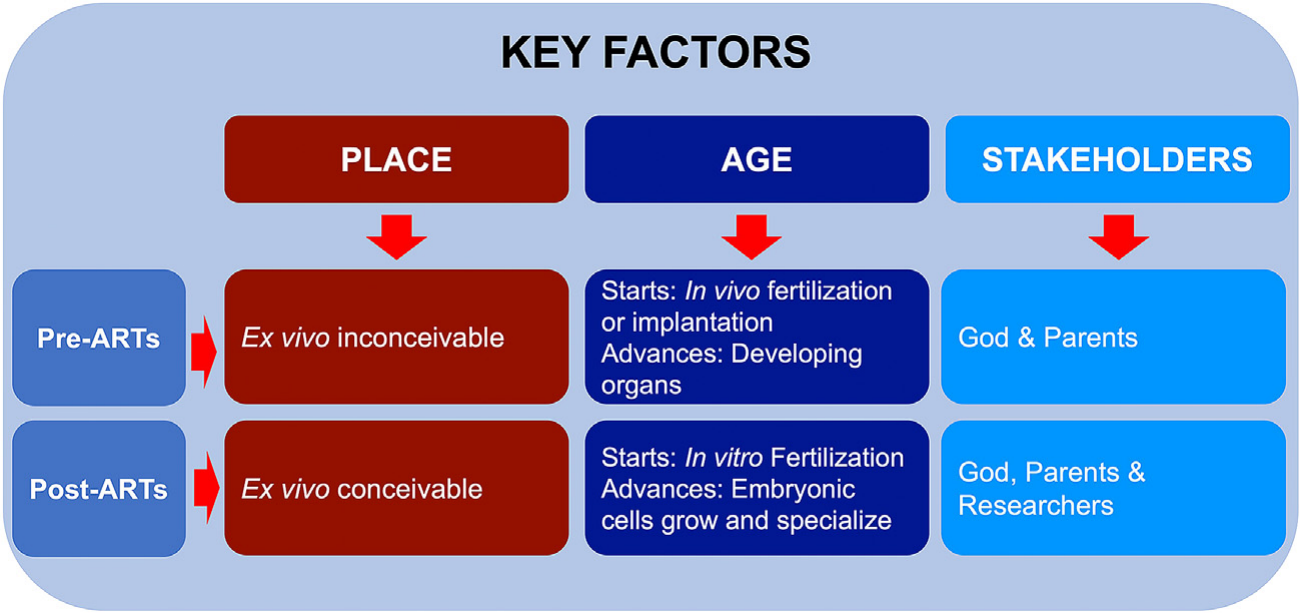
Key factors influencing the moral status of human embryos The moral standing of the human embryo is not static but would vary, depending on key factors whose perception is shaped by scientific advances: embryo’s place, age, and involved stakeholders. ARTs, assisted reproductive technologies.

Aligned with the foundational principles established in the classical deliberations and supported by the majority position advocated by the aforementioned institutions, we elaborate in the following paragraphs how the moral standing of the human embryo is contingent on a crucial and interconnected set of factors (Figure 1). Understanding these factors is key to comprehend why and how the reconsideration of the 14-day limit is compatible with the Islamic moral tradition.

#### Place

Contrary to prohibitionists, we argue that the moral status of *in vitro* embryos cannot be equated to that of *in vivo* embryos. Additionally, permissivists’ assertion that pre-implantation embryos lack any degree of dignity is untenable. While physically/genetically similar, the ethical distinction lies in their potential to develop into ensouled embryos and ultimately born children (Ghaly et al., 2020). Given current biomedical advancements, actualizing the human potential of *in vivo* embryos typically does not necessitate special efforts under normal circumstances, whereas *in vitro* embryos still require placement in the uterus and the management of the process of initiating pregnancy. Therefore, in the former case, realizing the potential that God created in *in vivo* embryos requires only human inaction or omission (*tark*). However, for *in vitro* embryos, *tark* alone is insufficient; active human intervention or commission (*fiʿl*) is necessary to achieve the same potential. This morally significant distinction between omission and commission has been applied in related bioethical discussions on end-of-life care issues (Ghaly, 2022). Concerning embryo models, as long as scientific evidence shows their inherent incapacity to develop into human persons, they should be accorded the same moral protection as biological materials, lacking even the dignity of a human embryo inside or outside the uterus.

#### Age

In the pre-assisted reproductive technology (ART) era, embryo age calculation commenced with events *inside* the uterus—fertilization or implantation—sometimes confused by classical religious scholars as concurrent occurrences. In the post-ART era, *ex vivo* fertilization could be “artificially” managed, and embryos could be stored for years. Since *ex vivo* implantation is unfeasible, calculating the age of embryo’s growth should start after fertilization. As the *ex vivo* embryo grows, it should attain higher dignity, aligning with pre-modern scholars’ discussions on the aforementioned concept of *takhalluq* (assuming human shape). Modern knowledge provided by genetics and genomics necessitates supplementing the traditional phenotype-based understanding of *takhalluq* with a genotype-based perception, beginning with the possession of a distinctive human genome. In essence, *in vitro* embryos undergo an initial genotype-based *takhalluq*, which later manifests into phenotype-based *takhalluq*, as *in vivo* embryos develop organs and limbs.

Essentially, *ex vivo* embryos occupy an intermediate category, lacking the dignity of either a zero-age or ensouled embryo. Embryo models, regardless of age, do not possess the dignity of a human embryo if they inherently lack the potential to develop into a human. However, if future scientific research demonstrates their capability to develop into a viable child, their dignity will then be regarded on par with similar *ex vivo* embryos.

Regarding the embryo’s maximum age for scientific research, contemporary discussions generally omit a specific age limit. However, the 120-day mark, associated with ensoulment, represents a critical threshold, beyond which most Muslim scholars would likely disapprove of embryo research. An earlier limit of 40 days would find support among almost all contemporary Muslim scholars, including those who hold that ensoulment occurs at that time or emphasize developmental milestones related to assuming human shape (*takhalluq*).

#### Stakeholders

In the pre-ART era, ethical considerations involved God, the Creator, necessitating His permission for any intervention, and the married couple contributing to the embryo’s makeup, requiring their consent. In the post-ART era, the scientific researcher emerges as a new stakeholder, as the embryo transitions into a research subject. The mainstream position, articulated in a famous fatwa issued in 2003 by the aforementioned IFC, permits embryo research under two main conditions: first, harvesting them from legitimate sources, like IVF surplus or religiously permissible miscarriage/abortion. Illegitimate sources include embryos created exclusively for research, therapeutic cloning, or those harvested from prohibited abortions. Second, parental consent is mandatory (Ghaly, 2012). This aligns with a fundamental principle that stems from Quranic verse 4:1, emphasizing that human embryos are created by God primarily to develop into human beings. Research can proceed if it does not supersede this principle, e.g., when valid reasons hinder this potential, like the nonviability of some *in vitro* embryos or developmental interruptions *in vivo*. For human induced pluripotent stem cells, consent from the individual, or appointed guardian, whose sample was utilized to generate the cells, is required. Two additional conditions are proposed here. Third, research must be justified on scientific and ethical grounds, approved by an ethics committee. Fourth, embryos should be altruistically donated, without financial transactions.

## Conclusion

Embryo models offer innovative avenues for studying human development and diseases, although ethical and safety concerns currently prohibit their transfer into animal or human uteri. While speculative, there is potential for genetically edited human stem cell-derived embryos to be transferred for infertility treatment or addressing genetic diseases. Universal guidelines restrict *in vitro* human embryo culture beyond 14 days, requiring jurisdictions to establish policies based on values and priorities. It is imperative for funding bodies, biomedical and bioethical societies, experts, and patient advocates to engage in international, cross-cultural discussions to foster consensus-building. Incorporating perspectives from world religions, including Islam, is crucial. We argue that a deeper understanding of diverse issues can enhance adherence to international guidelines, elevate public deliberation, foster trust, and improve practice standards. Global leaders in the field should actively include the voices of Muslim scientists and ethicists in discussions on the scientific and socio-moral implications of human embryo research. Regarding the Islamic moral tradition, we demonstrated that a perspective rooted in this tradition advocates for extending the 14-day limit, under certain conditions, to at least 40 days.
